# Clinical Significance of Statistical Differences

**DOI:** 10.36660/abc.20200006

**Published:** 2020-02

**Authors:** Luiz Maurino Abreu

**Affiliations:** 1 Hospital dos Servidores do Estado, Cardiologia, Rio de Janeiro, RJ - Brazil; 2 Estimulocor - Aval Clinica e Cardiológica, Rio de Janeiro, RJ - Brazil

**Keywords:** Diabetes Mellitus/complications, Risk Factors, Prevention and Control, Arrhythmias,Cardiac, Electrocardiography/methods, Data Interpretation Statistical

Diabetes is a serious chronic condition that occurs when your body cannot produce any or enough insulin or cannot effectively use the insulin it produces, resulting in high blood glucose levels. The so-called type 1 diabetes is caused by a set of genetic, environmental and autoimmune factors affecting insulin-producing pancreatic beta cells.^1^

Elevated blood glucose levels even below the diagnostic threshold of diabetes are associated with complications with the greatest impact on morbidity and mortality among people with diabetes.^2^ Systematic reviews involving 102 prospective studies in diabetic patients indicate that the relative risk of cardiovascular disease (CVD) is between 1.6 and 2.6, greater in younger people and slightly higher in women.^3^

Data published in the recent International Diabetes Federation (IDF Atlas) Atlas confirm that diabetes is one of the largest public health emergencies of the 21st century. By 2019 it is estimated that 468 million people have diabetes and projected to 578 million in 2030, and the alarming number of 700 million in 2045. The estimates are impressive: 135 million cases over 65 years of age; more than one million children and adolescents have type 1 diabetes; four million diabetes-related deaths in 2019 among 20- to 79-year-olds. All these numbers with a significant variation between the various regions of the world.^4^

The search for risk markers with the potential to predict diabetes-related adverse outcomes is constant. The high mortality risk of individuals with diabetes cannot be fully explained only by CVD or cardiovascular risk factors.^5^

The article by Inanir et al.^6^ in this issue retrospectively analyses electrocardiograms of patients with type 1 diabetes without comorbidities and without any medication except insulin, followed at the endocrinology outpatient clinic of Bolu City University Hospital, Turkey. Patients were compared to a control group of non-diabetic patients matched for age, sex, and body mass index, both groups younger than 45 years. The tracings were analyzed by two cardiologists blinded to the patients' conditions, focusing on the period of ventricular depolarization / repolarization with the various intervals and corrections for heart rate and the relationship with each other. The intervals QT, QTc, QTd, QTdc, Tp-e, JT, and Tp-e/QT, and Tp-e/QTc ratios were calculated. The correlation of these variables with disease duration and HgA1c value was also analyzed. There were statistically significant differences between the two groups with QTc of 412.9 ± 36 ms versus 384.2 ± 24.6 ms in diabetics compared with controls, respectively (p < 0.001), with a correlation with disease duration and HgA1c levels.

Several reports in the literature support that an increased QT interval represents a trigger for ventricular arrhythmias and even sudden death, with predictive value for all-cause mortality in diabetic and non-diabetic patients. However, QTc in a healthy population included in the Framinghan Heart Study was not predictive of cardiovascular death or sudden cardiac death. In another study, in healthy elderly, QTc > 450 ms in men and > 470 ms in women was an independent predictive risk factor for sudden cardiac death.^7,8^

The Polish Norwegian study (PONS), with a sample of 11,068 participants aged 45 to 64 years, distributed according to metabolic status, showed that the QTc interval progressively increased from those with normal blood glucose to those with impaired glucose tolerance, and was even higher in those with diabetes. The authors concluded that the results suggest that abnormal glucose metabolism affects ventricular repolarization regardless of other concomitant cardiovascular risk factors for diabetes.^9^

A literature review from 1990 assessing the association between prolonged QTc and risk of cardiovascular mortality and sudden death included seven prospective studies with 36,031 individuals, where 2,677 (8.7%) had QTc ≥ 440 ms. In this qualitative review, there was no definitive association between QTc interval and cardiovascular mortality or morbidity, except, without much consistency, in those with prior cardiovascular disease. The lack of coherence of the findings between the various subgroups underscores the likelihood that chance, bias, and/or confounding factors are plausible explanations for such disagreements. In addition, the small sample size in each study reduces power, and contributes to the inaccuracy of measurements given the technical challenges and follow-up time, among other uncontrolled factors. There are also variations among protocols used to obtain and analyze measurements in electrocardiographic tracings. When only one recording is used, the individual variability of the QTc interval over 24 hours is highlighted but drawing conclusions from one-time measurements about clinical events that usually occur years later ignores several other common factors in this population. The observation that patients with QTc interval ≥ 440 ms have a higher risk of total or cardiovascular mortality than those with lower values may reflect the role of QTc as a marker of still subclinical cardiovascular disease. In this analysis, none of the seven studies was designed to test the hypothesis that QTc interval is associated with total or cardiovascular mortality. Thus, uncontrolled confounders arising from uncollected or unknown variables may have influenced the results in either direction.^10^

QT dispersion (QTd) is defined as the difference between the longest and shortest QT intervals in any of the measured leads. The use of low number of leads was certainly the main cause of the lack of reproducibility repeatedly demonstrated in several studies that analyzed this parameter, making it difficult to determine its clinical significance especially in patients without known heart disease.^11^

Making use of technologies that allow for a larger number of derivations and recordings, with extended time, allowing the analysis of several records at different times is a trend in studies that explore the depolarization / repolarization periods. A study using the body surface mapping system, QT interval was measured in 80 unipolar chest leads, showing QTc 415.2+/-4.1 ms in DM 1 and 401.4+/-6.6 ms in controls (NS).^12^ The method allows the analysis of the electrocardiogram, the vetocardiogram and the mapping of depolarization / repolarization in more detail.^13^ The advantage of this procedure is its improved spatial sampling, allowing abnormalities that may be difficult to detect and measure using the 12-lead approach be better defined with the additional electrodes.

In the 1990s, at our Cardiology Service of the Hospital Federal dos Servidores do Estado, we had the opportunity to use the body surface mapping system in a sample of our population and the results were presented in a scientific conference held in Karlovy Vary, Czech Republic, where the method is quite used at Charles University, Prague. From this center there is an important review published in 2015 analyzing electrocardiographic changes in diabetes, revealing tachycardia, QRS and QT shortening, increased QT dispersion, reduced depolarization wave amplitude, reduced ventricular myocardial activation time, and T wave flattening confirmed by the lowest maximum and minimum value in the isopotential repolarization body surface maps. Most of these changes are even more pronounced in patients with cardiac autonomic neuropathy. Comparison with electrocardiographic changes in other diseases suggests that those present in patients with diabetes are not specific and are caused by an increase in sympathetic nervous system tone, which was indirectly confirmed by findings of heart rate variability in these patients.^14^

As we can see, obtaining measures of the depolarization/repolarization period has a long history and constant challenges. Some of the disparities in interpretation of findings arise from inconsistencies in the measurements obtained. The numerous factors involved in the inaccuracy of QT measurements and their repercussions as predictors in the clinical context make it difficult to assess the significance of minor QT changes even when they are statistically significant.

In summary, based on these observations, we cannot affirm that changes in depolarization / repolarization estimated by electrocardiography can be used as predictors of mortality and cardiovascular outcomes in the general population and apparently healthy diabetics. Further research is needed, which should include new technologies for serial measurements of these intervals and their measurement by a central electrocardiography laboratory. Such studies should be specifically designed to test the hypothesis that these depolarization / repolarization changes are associated with cardiovascular mortality
